# Multi-clonal evolution of multi-drug-resistant/extensively drug-resistant *Mycobacterium tuberculosis* in a high-prevalence setting of Papua New Guinea for over three decades

**DOI:** 10.1099/mgen.0.000147

**Published:** 2018-01-04

**Authors:** Arnold Bainomugisa, Evelyn Lavu, Stenard Hiashiri, Suman Majumdar, Alice Honjepari, Rendi Moke, Paison Dakulala, Grant A. Hill-Cawthorne, Sushil Pandey, Ben J. Marais, Chris Coulter, Lachlan Coin

**Affiliations:** ^1^​ Faculty of Medicine, University of Queensland, Brisbane, Australia; ^2^​ Institute for Molecular Bioscience, University of Queensland, Brisbane, Australia; ^3^​ Central Public Health Laboratory, Port Moresby, Papua New Guinea; ^4^​ Western Province Health Office, Western Province, Papua New Guinea; ^5^​ Burnet Institute, Melbourne, Australia; ^6^​ National Department of Health, Port Moresby, Papua New Guinea; ^7^​ School of Public Health, University of Sydney, Sydney, Australia; ^8^​ Queensland Mycobacterium Reference Laboratory, Pathology Queensland, Brisbane, Australia

**Keywords:** genomics, tuberculosis, mycobacteria, evolution, drug resistance

## Abstract

An outbreak of multi-drug resistant (MDR) tuberculosis (TB) has been reported on Daru Island, Papua New Guinea. *Mycobacterium tuberculosis* strains driving this outbreak and the temporal accrual of drug resistance mutations have not been described. Whole genome sequencing of 100 of 165 clinical isolates referred from Daru General Hospital to the Supranational reference laboratory, Brisbane, during 2012–2015 revealed that 95 belonged to a single modern Beijing sub-lineage strain. Molecular dating suggested acquisition of streptomycin and isoniazid resistance in the 1960s, with potentially enhanced virulence mediated by an *mycP1* mutation. The Beijing sub-lineage strain demonstrated a high degree of co-resistance between isoniazid and ethionamide (80/95; 84.2 %) attributed to an *inhA* promoter mutation combined with *inhA* and *ndh* coding mutations. Multi-drug resistance, observed in 78/95 samples, emerged with the acquisition of a typical *rpoB* mutation together with a compensatory *rpoC* mutation in the 1980s. There was independent acquisition of fluoroquinolone and aminoglycoside resistance, and evidence of local transmission of extensively drug resistant (XDR) strains from 2009. These findings underline the importance of whole genome sequencing in informing an effective public health response to MDR/XDR TB.

## Data Summary

1. The Illumina sequencing reads generated and analysed during the current study are available in the NCBI under the project file number PRJNA385247, https://www.ncbi.nlm.nih.gov/sra/?term=PRJNA385247

2. All scripts and command lines used for generation of structural variants are available, https://github.com/arnoldbaino/Daru_scripts

3. The file used for molecular clock analysis is available, https://figshare.com/s/73cfe6062e657419e084

**Impact Statement**

The Papua New Guinea government declared a multi-drug-resistant (MDR) tuberculosis (TB) emergency on Daru Island following excessive case numbers in recent years. Clinical experience suggested that most cases were epidemiologically linked, but to date limited molecular epidemiology studies have been performed. We utilized whole genome sequencing (WGS) to refine our understanding of the epidemiology and acquisition of drug resistance. We confirmed that the MDR TB outbreak on Daru Island is being driven by transmission of a modern Beijing sub-lineage strain. Molecular clock analysis of the dominant strain identified a long evolutionary history with four circulating clades. Clade C was dominant and included the majority of MDR strains, as well as a small cluster of recently evolved extensively drug-resistant (XDR) strains. Detailed characterization of resistance-conferring mutations provided novel insight into the underlying mechanisms of drug resistance and helped to elucidate putative compensatory or virulence genes. The insight gained by WGS provides added urgency to contain the spread of MDR and XDR TB transmission on Daru Island and beyond. It emphasized the need for early accurate diagnosis and increased access to new agents and regimens to improve patient outcomes.

## Introduction

Globally, an estimated 10.4 million cases of tuberculosis (TB) occurred in 2015 and TB caused by *Mycobacterium tuberculosis* (MTB) was the leading cause of death from a single infectious agent [1]. The emergence and spread of drug-resistant MTB strains pose a major challenge to global TB prevention efforts [2]. Multi-drug-resistant (MDR) TB, which is resistant to at least isoniazid and rifampicin, accounted for an estimated 480 000 new cases and 250 000 deaths in 2015 [1]. Additional resistance to fluoroquinolones and second-line injectables defines extensively drug-resistant (XDR) TB [3]. In MTB, drug resistance occurs mainly due to the accumulation of chromosomal resistance-conferring mutations without evidence of lateral gene transfer [4]. The emergence of drug resistance is dependent on the rate of acquisition of resistance-conferring mutations and the frequency with which these drug-resistant strains are transmitted, especially in communities with inadequate treatment or poor adherence to treatment [5, 6]. Compensatory mutations that limit the fitness cost imposed by drug resistance may enhance the clonal spread of the most successful drug-resistant strains [6–8].

Genotyping of MTB using the 24-locus mycobacterial interspersed repetitive unit (MIRU-24) remains widely used as a primary approach to strain characterization [9]. However, MIRU-24 interrogates small genomic regions that are susceptible to homoplasy and offers sub-optimal discriminatory power [10]. The discriminatory power of MIRU-24 is of particular concern with Beijing lineage strains [11]. Whole genome sequencing (WGS) offers optimal resolution to explore local transmission dynamics while also revealing the molecular mechanisms and evolution of drug resistance [6, 12, 13].

**Fig. 1.**
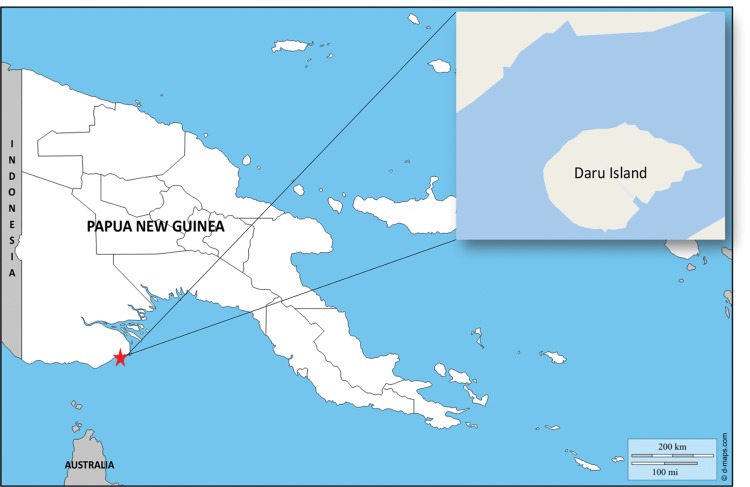
Map of Papua New Guinea illustrating the study location of Daru Island (inset). Daru Town is the capital of South Fly district, Western Province of Papua New Guinea. The original map was obtained fromhttp://d-maps.com

Papua New Guinea (PNG) has an estimated TB incidence of 432 per 100 000 population [1]. However, this reported incidence may be an underestimate because most rural areas are isolated with limited access to proper health services, and there are inaccuracies in census data and variations in population growth [14]. Daru Island (Fig. 1) is a 14.7-km^2^ island with a population of 16 714 [15, 16] and is one of the major hotspots for TB outbreaks [17]. The case notification rate in 2016 was estimated to be 2901 per 100 000 population (based on 485 cases notified) [15]. A recent national MDR TB survey showed that Daru Island had the highest concentration of MDR TB cases in PNG [17], with estimates of 0.76 % (125 cases) of the population diagnosed with drug-resistant TB in 2016 [15]. However, the MTB strains driving the epidemic on Daru Island, as well as the associated drug resistance mutations and temporal accrual of these mutations have not been described. Surveillance of underlying drug resistance mutations, in addition to routine phenotypic susceptibility testing, is important for optimizing TB treatment. In particular, it will help caution the appropriate use of the latest as well as re-purposed antibiotics to maximize their effectiveness. Understanding the evolution of drug resistance mutations is important for evaluating the strengths and weaknesses of public health responses.

## Methods

We performed a retrospective assessment of all clinical isolates referred from Daru General Hospital to the Supra-National Referral Laboratory (SRL) in Brisbane, Australia, over a 29.5-month period from 1 October 2012 to 15 March 2015, which had a positive *M. tuberculosis* culture. Clinical specimens are routinely referred to SRL for culture and drug sensitivity testing. The criterion for referral is detection of rifampicin resistance by Xpert MTB/RIF assay (Cephid) on concurrent samples or at the individual discretion of a clinician.

MIRU-24 and WGS were performed to understand the genetic diversity of circulating strains, reveal the mutations associated with drug resistance and explore the evolution of these mutations.

### Study setting and specimen selection

Daru Town, located on Daru Island, is the provincial capital of PNG’s Western Province and has an estimated population of around 16 714 [16]. Daru General Hospital provides healthcare services to residents of Daru Island and the surrounding mainland communities in South Fly District.

### Study procedures

DNA was extracted from cultures with confluent growth on Löwenstien-Jensen (LJ) slopes using a Spin column High pure PCR template prep kit (Roche Diagnostics). MIRU-24 genotyping was performed as per standard protocols [18], and GeneMapper version 4.0 (Applied Biosystems) was used for fragment analysis.

Phenotypic drug susceptibility to the first-line drugs rifampicin (1.0 µg ml^−1^), isoniazid (0.1 µg ml^−1^, low-level; 0.4 µg ml^−1^, high-level), streptomycin (1.0 µg ml^−1^), ethambutol (5.0 µg ml^−1^) and pyrazinamide (100 µg ml^−1^) was performed based on the proportion method using the automated Bactec Mycobacterial Growth Indicator Tube (MGIT) 960 system (Becton Dickinson). For MDR TB isolates, susceptibility to the second-line drugs amikacin (1.0 µg ml^−1^), capreomycin (2.5 µg ml^−1^), kanamycin (2.5 µg ml^−1^), ethionamide (5.0 µg ml^−1^), ofloxacin (2.0 µg ml^−1^), *p*-aminosalicylic acid (4.0 µg ml^−1^) and cycloserine (50 µg ml^−1^) was determined using the MGIT system [19, 20].

DNA for WGS was isolated using a lysozyme-phenol chloroform-based method and DNA was purified using a QIAamp DNA mini prep kit (Qiagen). Paired end libraries were prepared using an Illumina Nextera XT DNA library preparation kit and sequenced using the Illumina MiSeq sequencing platform at Westmead Institute for Medical Research and Australia Genome Research Facility (Sydney, Australia). Raw reads were used to derive octal codes using *in silico* SpolPred [21] and spoligotypes were inferred using the international database (SpolDB4) [22]. Paired end reads were checked for quality using FastQC v0.11.2 [23], and trimmomatic v0.27 [24] was used to remove low-quality base pairs (Phred score <30) especially at 3′ ends. Trimmed reads were mapped to the *H37Rv* reference genome (GenBank: NC_000962.3) using BWA-mem with default settings [25]. The mean reference coverage was 98.4 % (range 96.4–99.8 %) and mean high-quality base coverage was 70.7× (range 25–182×).

GATK UnifiedGenotyper was used to call SNPs and small indels [26]. SNPs and small indels with at least 10× read depth, 80 % allele frequency and with at least 10 bp difference between neighbouring SNPs/indels were retained. Indels of greater than four mutations were excluded from the analysis unless occurring within a known drug resistance-associated gene. High-quality SNPs and indels were annotated using SnpEff v4.1 [27] and those in repetitive regions such as proline glutamine/proline proline glutamate family genes (PE/PPE) were excluded from analysis. We characterized mutations in known genes (including regulatory mutations) that confer resistance to rifampicin, isoniazid, ethambutol, streptomycin, pyrazinamide, fluoroquinolones, amikacin, capreomycin, kanamycin, ethionamide, *p*-aminosalicyclic acid, cycloserine, bedaquiline, linezolid and delamanid according to a literature review (Table S1, available in the online version of this article).

### Allelic diversity, phylogeny and molecular dating

The clonal structure from MIRU-24 was inferred using a minimum spanning tree algorithm implemented in Bionumerics v6.7 (Applied Maths). Data sets from two previous independent WGS studies, PRJEB7281 and PRJEB2358 [28], were used as MTB global representatives in phylogenetic analysis.

Concatenated SNP alignment was used to construct a maximum-likelihood phylogenetic tree using RAxML v7.4.2, the GTRCAT model with 1000 bootstrap replicates [29] and visualized using FigTree v1.4.2.

Molecular dating of the Beijing outbreak cluster was first performed by Beauti using an alignment containing both invariable and variable sites as an input file for beast v1.8.2 [30]. Base substitution was modelled using the Hasegawa-Kishino-Yano (HKY) or General Time Reversible (GTR) model with an estimated base frequency and a gamma distribution among-site rate variation with four rate categories. The lognormal relaxed clock (uncorrelated) model which assumes independent mutation rates on different branches was used [12, 13, 31, 32]. The tree was calibrated using date of sample collection as tip dates for each genome, specified in years before the present.

We used a uniform prior distribution for all the trees using a mean mutation rate of 0.35 SNP/genome/year [12, 33–35] and compared the model performance under the different demographic models by calculating the Bayes factors from marginal likelihood estimates obtained from path sampling/stepping stone sampling [36]. For each analysis, two independent runs of 50 million steps using the Markov chain Monte Carlo (MCMC) method were performed, discarding 10 % burn-in and drawing samples every 5000 steps. Three independent runs of the best model were performed for consistency. Tracer was used to examine Markov chain convergence, adequate mixing, chain length and effective sample size (ESS >200). TreeAnnotator was used to obtain the best supported topology under the maximum clade credibility method.

A constant demographic model that used GTR substitution and gamma distribution at four categories was found to be the best fit since it had a better marginal likelihood estimate compared to the other models (Table S2). The overall mutation rate was estimated to be 0.36 SNP/genome/year [95 % highest posterior density (HPD) 0.24–0.48], which is consistent with other published reports [33, 37, 38].

Resistance-conferring mutations were parsimoniously mapped on the phylogenetic tree (considering no reversion) and divergence time was used to infer the likely timing of drug resistance acquisition. The number of strains that shared known resistance-conferring SNPs from the tree nodes (Fig. 3) was enumerated to infer transmitted drug resistance-conferring versus non-shared drug resistance-conferring SNPs (tree branches) to infer acquired drug resistance [6, 13, 39]. SeqTrack was used to create transmission networks among the clades. A Fisher’s exact test in R statistical package was used to assess the association of observed drug resistance mutations and MDR/XDR phenotypes.

## Results

### Global phylogeny

**Fig. 2.**
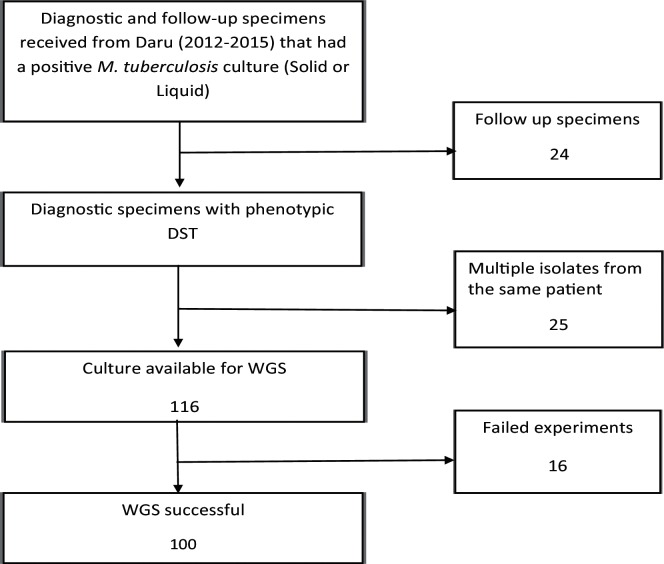
Workflow to derive clinical isolates included in the study collected between 1 October 2012 and 15 March 2015. PNG, Papua New Guinea; SRL, Supra-National Reference Laboratory; DST, drug susceptibility test; WGS, whole genome sequencing.

We characterized 100 out of 165 isolates collected during 2012–2015 from Daru, PNG, by MIRU-24 and WGS (Fig. 2). MIRU-24 analysis revealed that 95 isolates formed a single dominant cluster (Fig. S1). Analysis of SNPs derived from WGS revealed all the strains in the dominant cluster formed a monophyletic clade in the East-Asian lineage (*sensu stricto* modern Beijing lineage), while all the remaining strains belonged to the Euro-American lineage (Fig. S2). According to the classification of Coll *et al.* [40], the modern Beijing lineage was revealed to be Beijing sub-lineage 2.2.1.1 (Table S3). The Beijing sub-lineage had a median of 23 pairwise differing SNPs between samples (range 0–62), highlighting its limited genetic diversity (Fig. S3). This lineage was separated by at least 32 SNPs from the nearest neighbouring modern Beijing genome included in the representative phylogenetic tree (Table S4).

### Molecular dating and drug resistance

**Fig. 3.**
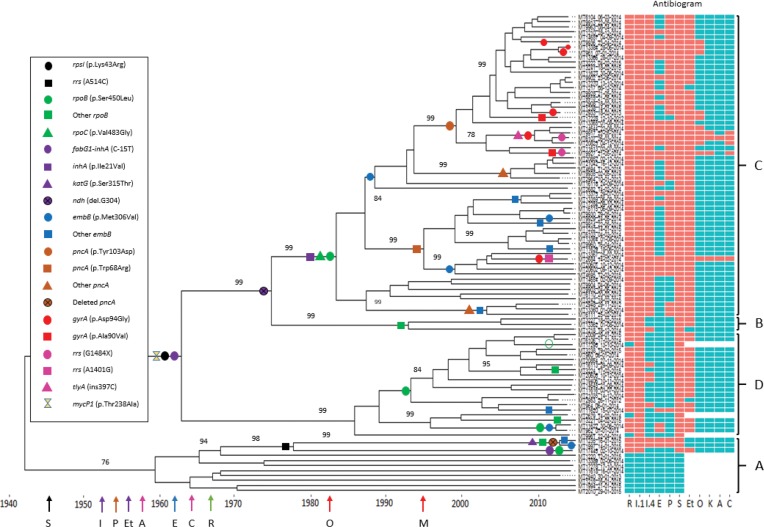
Phylogeny of Beijing sub-lineage strains with dated acquisition of drug resistance mutations. With four clades (A–D) recognizable, coloured shapes representing fixed resistance-conferring mutations for a TB drug or class of TB drug and putative mutations (*ndh* and *mycP1*) mapped on to tree branches parsimoniously assuming no reversion. Small red circle, *gyrA* (p.Asp94Ala); open green circle, no *rpoB* mutation. On the *x*-axis are coloured arrows representing the year of drug discovery: black, S (streptomycin, 1946); green, R (rifampicin, 1966); purple, I (isoniazid, 1952) and Et (ethionamide, 1956); blue, E (ethambutol, 1961); brown, P (pyrazinamide, 1954); red, O (ofloxacin, 1982) and M (moxifloxacin, 1996); pink, A (amikacin, 1957) and C (capreomycin, 1963) while antigiobram represents *in vitro* susceptibility: red squares, resistant; blue squares, susceptible. Bootstrap support values are shown where they exceed 70 %.

Table 1 summarizes known and new putative drug resistance mutations detected within the Beijing sub-lineage, which were parsimoniously mapped to a molecular clock phylogenetic tree that identified four clades: A (*n*=11), B (*n*=3), C (*n*=60) and D (*n*=21) (Fig. 3). Strains in clade A were the most distantly related and accumulated the fewest drug resistance mutations. Clade C demonstrated massive clonal expansion and included the majority of the MDR strains (54 MDR and six XDR). Ten SNPs, including a mutation in *mycP1* (p.Thr238Ala), differentiated clades B, C and D from clade A (Table S5; polymorphisms that are specific to Beijing lineage outbreak clusters clade B, C and D).

**Table 1.** Drug resistance mutations and phenotypic drug resistance observed among Beijing sub-lineage strains Aminoglycosides: A, amikacin; K, kanamycin; C, capreomycin. Isoniazid, all samples with phenotypic resistance had one of the mutations listed; ethambutol, 27/54 samples with one of the listed mutations were phenotypically susceptible. Known mutations to bedaquiline, delamanid, linezolid and clofazimine genes were not identified.

TB drug	Gene	Mutation	Phenotypically resistant (*n*)	Phenotypically susceptible (*n*)	Critical concentration (µg/ml)
Rifampicin	*rpoB*	Ser450Leu*	78	0	1
		Asp435Val	2	0	
		Asp435Tyr	2	0	
		His445Arg	1	0	
		Ile480Val*	1	0	
		His445Asp	1	0	
	*rpoC*	Val483Gly	60	0	
		lle491Thr	1	0	
	*rpoA*	Val183Gly	1	0	
Isoniazid	*inhA*	Ile21Val	60	0	0.4
	*fabG1-inhA*	C15T	65	0	0.4
			20	0	0.1
	*katG*	Ser315Thr	2	0	0.4
	*ndh*	del, G304†	61	0	0.4
			2	0	0.1
Ethambutol	*embB*	Met306Val*	23	21	5
		Met306Ile	1	1	
		Gly406Ala	0	1	
		Gln497Arg*	2	5	
Pyrazinamide	*pncA*	Tyr103Asp	27	1	100
		Trp68Arg	17	0	
		Thr135Pro	1	0	
		Gln10Pro	3	0	
		Asp12Glu	5	0	
		Del, gene†	2	0	
Streptomycin	*rpsL*	Lys43Arg	84	0	8
	*rrs*	A514C	3	0	
Ethionamide	*fabG1-inhA*	C15T	80	2	10
Quinolones	*gyrA*	Ala90Val	2	0	2.5
		Asp94Ala	1	0	
		Asp94Gly	8	0	
Aminoglycosides	*rrs*	A1401G	1 (A,K,C)	0	4
		G1484A	1 (K,C)	1 (A)	
		G1484T	1 (A,K,C)	0	
	*tlyA*	ins397C	3 (C)	1 (C)	

*Two dual mutations (Ile480Val and Ser450Val, and Gln497Arg and Met306Val).

†Putative mutations.

Strains in clades B, C and D demonstrated universal streptomycin resistance conferred by an ancestral *rpsL* (p.Lys43Arg) mutation acquired in the 1960s. The strain ancestral to clades B, C and D also acquired an *inhA* promoter mutation (*fabG1-inhA*, C-15T) associated with low-level isoniazid resistance in the 1960s. The same *inhA* promoter mutation occurred independently in a single clade A strain, while some (3/11) clade A strains acquired streptomycin resistance conferred by an *rrs* (A514C) mutation. Clade C displayed universal high-level isoniazid resistance potentially conferred by an intragenic *inhA* mutation (p.Ile21Val) acquired in the 1980s. Occasional high-level isoniazid resistance in clade B (1/3) and clade D (2/21) strains was not associated with an *inhA* coding mutation while a *katG* (p.Ser315Thr) mutation was detected in only two clade A strains.

Of the 84 isoniazid-resistant strains tested for ethionamide resistance, 80 (95.2 %) were ethionamide co-resistant (comprising 64/67 high-level and 16/17 low-level isoniazid-resistant strains). Of the four remaining ethionamide-susceptible strains resistant to isoniazid, two have a *katG* mutation but no *inhA* coding or promoter mutations. All strains with co-resistance to at least low-level isoniazid and ethionamide had a *fabG1-inhA* mutation. We explored the co-occurring mutations observed in these strains (Table 2). All strains with co-occurring *fabG1-inhA*, *inhA* (p.Ile21Val) and *ndh* (del.G304) mutations were ethionamide-resistant, while 2/3 with both *fabG1-inhA* and *ndh* mutations were ethionamide-resistant. No other mutations associated with ethionamide resistance, such as *ethA* or *ethR*, were observed.

**Table 2.** Association of isoniazid and ethionamide phenotypes and common genes involved in resistance among Beijing sub-lineage strains INH, isoniazid; ETH, ethionamide. A total of 64/67 strains with high-level INH resistance were ethionamide-resistant. Note that three strains (*fabG–inhA* only) with low-level isoniazid resistance were not tested against ethionamide as they were not MDR.

Gene combinations*	Total	High-level INH resistant	Low-level INH resistant	Ethionamide resistant	Ethionamide susceptible
*fabG1-inhA+inhA+ndh*	60	60	0	60	0
*fabG1-inhA+ndh* only	3	1	2	2	1†
*fabG1-inhA* only	22	4	18	18	1
*katG* only	2	2	0	0	2
*inhA* only	0	0	0	0	0
*ethA* only	0	0	0	0	0
*ethr* only	0	0	0	0	0

*Observed mutations are in Table 1.

†High-level resistance to INH.

Multi-drug resistance was first acquired by clade C in the 1980s with acquisition of a frequently encountered *rpoB* mutation (p.Ser450Leu), together with a compensatory *rpoC* mutation (p.Val483Gly). The ancestral strain in clade B acquired a different *rpoB* mutation (p.His445Arg) in the 1990s without a compensatory mutation. One rifampicin-resistant clade A strain acquired a different *rpoC* compensatory mutation (p.Ile491Thr). The majority of clade D strains (17/21) also acquired the p.Ser450Leu *rpoB* mutation, but at a later time point and only one strain had a compensatory *rpoA* (p.Val183Gly) mutation. One rifampicin-resistant clade D strain had two *rpoB* mutations (p.Ser450Leu and p.Ile480Val), as confirmed by sequence reads spanning both mutations (Fig. S4). One rifampicin-susceptible isolate in clade D was found to be part of a rifampicin-resistant clone carrying a common *rpoB* (pSer450Leu) mutation although we did not observe this mutation in this isolate despite adequate sequence coverage (Fig. S5).

There was good correlation between genotypic mutations and phenotypic drug susceptibility (Table 1). However, only 50 % (27/54) of the strains with putative ethambutol resistance-conferring mutations were phenotypically resistant at the critical concentration (5.0 mg l^−1^); MIC testing to encompass lower concentrations of ethambutol was not performed. The *embB* (p.Met306Val) mutation first occurred in clade C during the 1980s with independent acquisition at later time points in clades A, C and D. Another *embB* mutation (p.Gln497Arg) occurred independently on two occasions in clade C.

The majority of clade C (53/60; 88.3 %) strains had pyrazinamide resistance mutations. The earliest mutation *pncA* (p.Trp68Arg) was acquired in the 1990s. The only two pyrazinamide-resistant clade A strains shared a 3990-bp genomic deletion (position 2 287 064–2 291 054, Fig. S6) spanning four genes including *pncA*.

### XDR TB genotypes

Fluoroquinolone resistance due to mutations in the *gyrA* gene was detected in 11/60 (18 %) clade C strains; six were XDR TB (Table 1). A single four-member XDR clone emerged around 2009, characterized by an additional capreomycin resistance mutation (*tlyA*, insertion C.397). One XDR strain with phenotypic resistance to amikacin, kanamycin and capreomycin had both *rrs* (G1484T) and *tlyA* (insertion 397C) mutations, while another with an *rrs* (G1484A) mutation was phenotypically only resistant to kanamycin and capreomycin (Fig. S7). We utilized the arrangement of drug resistance mutations on the tree (Fig. 3) together with transmission network analysis of the large clades C and D (Fig. S8) to determine whether MDR/XDR TB emerged because of acquired resistance or direct transmission. Transmitted drug resistance to isoniazid, rifampicin, ethambutol and streptomycin was evident in clades B, C and D (*P*<0.0001, Table 3). From transmission network analysis, six and four distinct clusters (2–4 isolates per cluster) that are tightly linked (three or fewer SNP differences between samples within a cluster) were identified in clades C and D, respectively (Fig. S8). For the remaining isolates within the clades, 23 clade C strains (included four XDR) and six clade D strains were within 3–5 SNP differences to at least one other isolate reminiscent of transmission.

**Table 3.** Frequency of transmitted (cluster) and acquired (independent) drug resistance mutations identified in different clades of the Beijing sub-lineage strains inferred from Fig. 3

		Clade A		Clade B	Clade C		Clade D	
		Independent	Cluster	Cluster	Independent	Cluster	Independent	Cluster
Locus	*rpoB*	1	2	3	0	60	3	16
	*fabG1-inhA*	1	0	3	0	60	0	21
	*inhA*	0	0	0	0	60	0	0
	*katG*	0	2	0	0	0	0	0
	*rpsl*	0	3	3	0	60	0	21
	*embB*	2	0	0	3	46	1	2
	*pncA*	0	2	0	0	53	0	0
	*gyrA*	0	0	0	7	4	0	0
	*rrs*	0	0	0	3	0	0	0
	*tlyA*	0	0	0	0	4	0	0

## Discussion

We provide the first insight into the molecular basis of drug resistance and genealogy of the dominant strains circulating in a high-prevalence setting on Daru Island in the Western Province, PNG. Our findings indicate that the large number of MDR TB cases is driven by the transmission of a highly drug-resistant cluster of a modern Beijing lineage strain. Studies from other parts of PNG have demonstrated dominance of Euro-American lineage strains, [20, 41] although the contribution of Beijing sub-lineage strains may have been underestimated. We cannot comment on the geographical spread of the Beijing sub-lineage, but phylogenetic analysis combined with detailed molecular dating suggests that it has been in local circulation since the 1940s and first acquired drug resistance mutations in the 1960s. The identification of four different clades with distinct evolutionary trajectories suggests a ‘permissive’ environment for MTB strains to acquire and spread drug resistance within the study setting.

Clade C was the most successful clade, acquiring resistance to all four first-line drugs, and demonstrated clonal spread of both MDR and XDR strains. Phylogenetic analysis indicated that isoniazid and streptomycin resistance were acquired by strains ancestral to clades B, C and D. This is consistent with the use of non-rifampicin-containing regimens in the 1960s that were highly reliant on isoniazid and streptomycin [42]. Similar analysis in Russia and South Africa where large MDR outbreaks have been recorded also indicated that streptomycin and isoniazid resistance were acquired before the introduction of rifampicin-containing regimens [6, 12]. These settings mostly reported high-level isoniazid resistance due to *katG* mutations. In a recent multinational study, *katG* (p.Ser315Thr) was identified as the harbinger mutation that is most frequently associated with future MDR occurrence and risks subsequent clonal spread [39].

Other settings with strains possessing the signature *fabG1-inhA* (C-15T) mutation that confers low-level isoniazid resistance with ethionamide co-resistance [43] have been observed in South Africa [44] and Portugal [45], although the Lisbon strain was of the LAM family. Phenotypic drug susceptibility results of clade C strains demonstrated universal high-level isoniazid resistance, which might be explained by the accumulation of an additional *inhA* (p.Ile21Val) mutation. The double accumulation of mutations may confer high-level isoniazid resistance without the fitness cost associated with *katG* mutations, supporting successful clonal expansion and acquisition of additional resistance-conferring mutations to other drugs [46]. An additional consideration is the possibility that the *ndh* frame shift mutation (deletion of Glu102fs) observed only in clades B and C may also have contributed, potentially as a compensatory mutation, because SNPs in the *ndh* gene have been associated with increased intracellular NADH/NAD+ ratios, and hence competitive inhibition of activated isoniazid [47]. With the recognized description of mutations that confer resistance to isoniazid and ethionamide, we anticipate that introduction of a short course regimen for MDR TB in this setting may be ineffective. This is because the dominant drug-resistant strain has underlying high-level resistance to two of six regimen drugs (isoniazid and proethionamide-ethionamide analogue) due to coexistence of *inhA* promoter and *inhA* gene mutations.

The selective advantage of clade C might have been enhanced further by the acquisition of a classic *rpoB* (p.Ser450Leu) rifampicin resistance determining mutation together with a compensatory *rpoC* (p.Val483Gly) mutation that abrogates its negative fitness effects [7]. Similar to previous observations, [48] one strain had two *rpoB* mutations, of which p.Ile480Val, which lies outside the 81-bp rifampicin resistance determining region, may represent a compensatory mutation. The single strain within a clade D *rpoB* (p.Ser450Leu) cluster, in which we were unable to detect an *rpoB* mutation (75× sequence depth) even though all neighbouring strains had the same mutation, may have been due to reversion that might have occurred after a transient mutator phenotype. Schmalstieg *et al.* attributed this ‘unstable or transient state’ event to be caused by sub-therapeutic drug exposures and effects of efflux pumps especially in the first steps of acquiring higher level resistance [49]. This has never been observed in rifampicin resistance and further investigation into the functionality of this aspect will help to determine its effects on the development of resistance and transmission.

An effective host immune response is critical to protect individuals against TB and to limit ongoing transmission within communities. We identified a putative non-synonymous *mycp1* (p.Thr238Ala) mutation that is ancestral to clades B, C and D. Mouse models used to identify MTB ESX-1 (ESAT-6 secretion system 1) substrates and their effects on host cells found *mycP1* proteins to be essential for early replication in macrophages and contribution to virulence by allowing escape of mycobacteria from the phagosome into the cytosol of infected macrophages [50, 51]. Site-directed mutagenesis that inactivated *mycP1* increased the expression of ESAT-6. Altered host immune responses effected by the putative *mycp1* (p.Thr238Ala) mutation may have increased the virulence and transmissibility of the Beijing sub-lineage strain because the observed mutation is within the active site [52], but this remains speculative as clade B demonstrated limited clonal expansion.

Previous studies have found a strong correlation between MDR TB and resistance to pyrazinamide or ethambutol, which highlights the problem of drug resistance amplification with continued use of first-line treatment regimens in patients with undiagnosed MDR TB [53, 54]. Our study provides evidence that streptomycin resistance in this setting is long standing and, as such, probably provided little protection against the development of resistance to first-line oral drugs when used in the World Health Organization (WHO)-endorsed retreatment regimen, since it is potentially compromised by prior treatment.

There was good correlation between genotypic and phenotypic drug resistance profiles, except for ethambutol. This reflects the complex genetic basis of ethambutol resistance, but also emphasizes the highly variable results achieved with phenotypic drug susceptibility testing [13]. Safi *et al*. demonstrated the epistatic nature of ethambutol resistance by establishing how mutations in *embB* are often accompanied by polymorphisms in other genes such as *nuoD* and *Rv3806c* and hence lead to progressive increases in the critical concentration [55]. We could not identify any of the accompanying mutations previously identified, but hypothesize that a reduction in the critical concentration used may improve the correlation between resistance phenotype and genotype.

The manifestation of transmitted MDR TB and minimal transmission of XDR TB highlights a problem with preventing transmissions. There is need for rapid access to reliable drug susceptibility testing (DST) and increased timely access to new and repurposed agents such as bedaquiline, delamanid and linezolid for effective management of MDR/XDR TB. Controlled clinical trials performed in other endemic settings such as Russia [56] before roll out of regimens with newer drug agents are required for establishment of the minimum number of drugs and duration of treatment that constitute an effective regimen. No putative resistance mutations were detected which have been implicated in resistance to newer agents. Mutations at *rrs* position 1484 may affect aminoglycoside susceptibility differently, since one XDR sample with an *rrs* (G1484T) mutation had phenotypic amikacin resistance, while another with an *rrs* (G1484A) mutation was susceptible to amikacin. In a systematic review of studies that assessed the use of second-line injectables, the *rrs* (G1484T) mutation was described as a specific predictor of injectable drug resistance but not the *rrs* (G1484A) mutation [57]. The Hain MTBDRsl line probe assay recently endorsed by WHO for rapid detection of second-line drug resistance has a probe for *rrs* G1484T but may detect G1484A as a failure of wild-type binding [58] and hence incorrectly infer resistance to all second-line injectables, which could lead to an incorrect choice of treatment.

Our study is limited by its retrospective nature and the fact that the clinical isolates tested failed to capture the whole population with MDR TB on Daru Island. Although we sequenced all available isolates, unreliable transport of specimens to the reference laboratory resulted in an incomplete sampling of MDR TB during the study period, leading to a modest sample size. Due to the modest sample size and short study period we chose to use a fixed mutation rate, which may have reduced the accuracy of evolutionary time point estimates. Another limitation is the use of sample collection date rather than date of diagnosis, although exclusion of follow-up samples should limit the impact of this on evolutionary analysis. Despite these limitations, our study represents the largest drug resistance collection from PNG described so far and provides an unbiased view of the evolution of MDR TB on Daru Island. The estimated dates of divergence are biologically plausible and are temporally consistent with respect to introduction of anti-TB drugs on the global market. Unfortunately a detailed description of when specific drugs were first used in the study setting was unavailable, although culture and drug susceptibility testing were started in 2010. Finally, we lacked clinical and epidemiological information to enhance our genomic analysis. This information is critical to identify individuals and population characteristics that facilitate ongoing transmission of drug-resistant strains.

WGS confirmed both recent transmission of drug-resistant TB and repeated acquisition of drug resistance mutations, driven by a modern Beijing sub-lineage strain. A major concern is that further spread of the strain to adjacent geographical areas, including the capital city, Port Moresby, may amplify transmission within PNG. There is urgent need for improved early detection of drug-resistant TB cases with linkage to effective care programmes in order to limit drug resistance amplification and terminate ongoing transmissions.

## Data bibliography

1. NCBI under project file number PRJNA385247 (2017)
2. NCBI under project file number PRJEB7281 (2014)
3. NCBI under project file number PRJEB2358 (2011)

## Supplementary Data

787 Supplementary File 1
