# Supplementary material for: Multi-clonal evolution of multi-drug-resistant/extensively drug-resistant Mycobacterium tuberculosis in a high-prevalence setting of Papua New Guinea for over three decades
Source: Microb Genom. 2018 Jan 4;4(2):e000147. doi: 10.1099/mgen.0.000147 (PMC5857374; doi:10.1099/mgen.0.000147)
Supplement: Supplementary File 1 [file mgen-4-147-s001.pdf]

Figure S1: MIRU-24 minimum spanning tree of *M. tuberculosis* isolates collected from Daru Island, Papua New Guinea constructed using Bionumerics v6.7. Sectioned circles represent two or more isolates that share identical allele profiles. Values on the branch*es* represent allelic difference between isolates. Green-dominant cluster (244252352644425163353824), Red-minor cluster, Purple-unique isolates

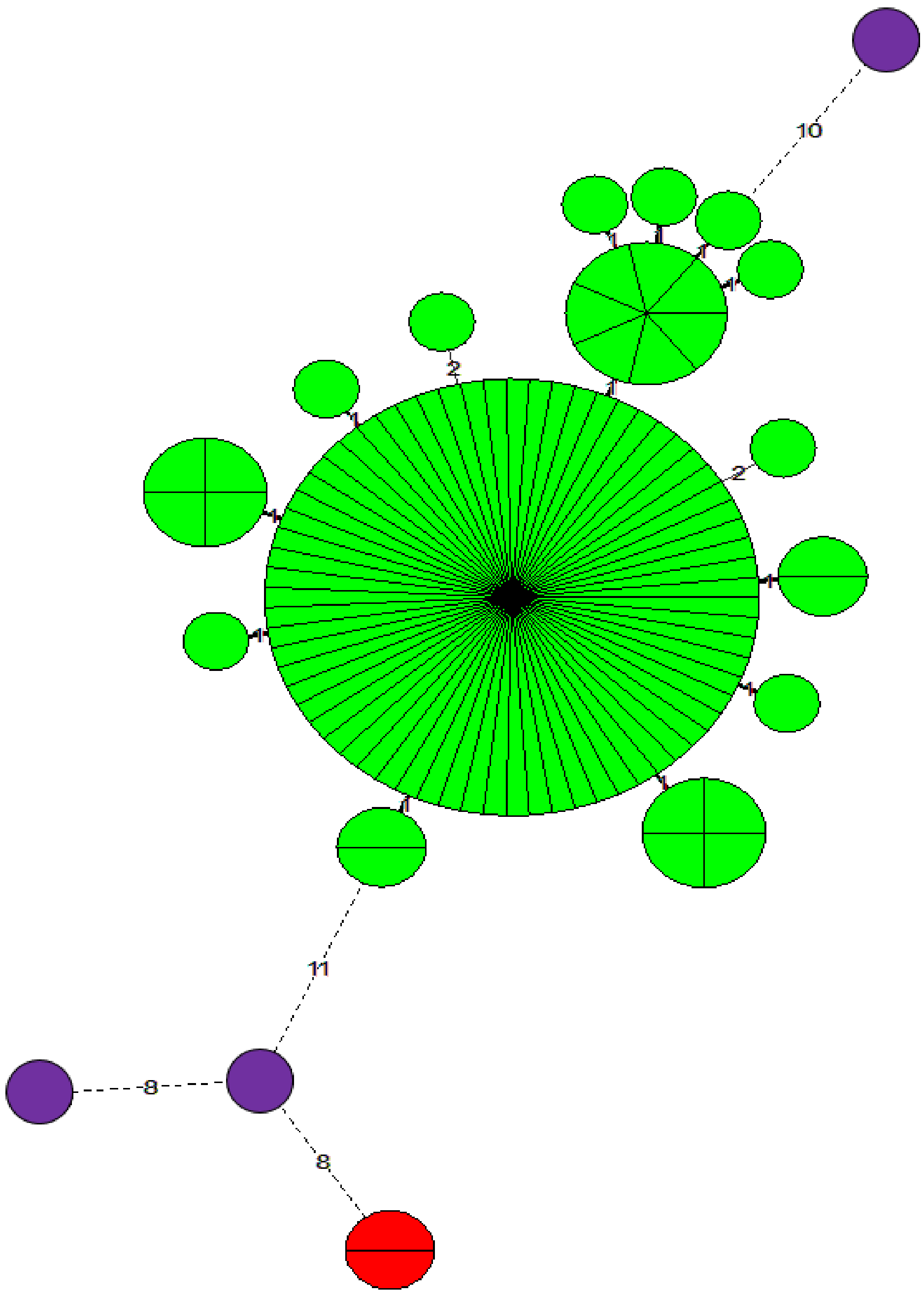

Table S1: list of some known resistance conferring genes and compensatory genes

	Drugs															
	**RIF**	**INH**	**ETB**	**PZA**	**STM**	**ETH**	**OFX**	**AMK**	**KAN**	**CAP**	**DCS**	**PAS**	**BDQ**	**LZD**	**DLM**	**CFM**
**Genes**	*rpoB*	*inhA* promoter	*embB*	*pncA*	*rpsL*	*fabG-inhA*	*gyrA/gyrA**	*rrs*	*eis* promoter	*tlyA*	*alr*	*ribD*	*atpE*	*rrl*	*fgd1*	*Rv0678*
	*rpoC**	*inhA*	*embC*	*rpsA*	*gidB*	*ethA*	*gyrB*	*whiB7*	*whiB7*	*whiB7*	*ddl*	*thyA*	*Rv0678*	*rplC*	*ddn*	*Rv1979c*
	*rpoA**	*katG*	*embA*	*panD*	*rrs*	*ethR*		*gidB*	*rrs*	*rrs*	*cycA*	*dfrA*			*fbiA*	*Rv2535c*
		ndh	*ubiA*						*gidB*	*gidB*		*folC*			*fbiB*	
		furA	*embR*												*fbiC*	
		oxyR	*iniA*													
		aphC	*iniC*													
		fadE24	*manB*													
		srmR														
		kasA														
		mshA														

Resistance conferring genes for RIF-rifampicin, INH-isoniazid, ETB-ethambutol, PZA-pyrazinamide, STM-streptomycin, ETH-ethionamide, OFX-ofloxacin, AMK-amikacin, KAN-kanamycin, CAP-capreomycin, DCS-cycloserine, PAS-para-aminosalicyclic acid, BDQ-bedaquiline, LZD-linezolid, DLM-delamanid, CFM-clofazimine

*Known compensatory genes

Publications used to identify these genes include

1. Ramaswamy, S. V., Dou, S. J., Rendon, A., Yang, Z., Cave, M. D., & Graviss, E. A. (2004). Genotypic analysis of multidrug-resistant Mycobacterium tuberculosis isolates from Monterrey, Mexico. *J Med Microbiol, 53*(Pt 2), 107-113. doi: 10.1099/jmm.0.05343-0
2. Bloemberg, G. V., Keller, P. M., Stucki, D., Trauner, A., Borrell, S., Latshang, T., . . . Bottger, E. C. (2015). Acquired Resistance to Bedaquiline and Delamanid in Therapy for Tuberculosis. *N Engl J Med, 373*(20), 1986-1988. doi: 10.1056/NEJMc1505196
3. Comas, I., Borrell, S., Roetzer, A., Rose, G., Malla, B., Kato-Maeda, M., . . . Gagneux, S. (2012). Whole-genome sequencing of rifampicin-resistant Mycobacterium tuberculosis strains identifies compensatory mutations in RNA polymerase genes. *Nat Genet, 44*(1), 106-110. doi: 10.1038/ng.1038
4. Jnawali, H. N., Yoo, H., Ryoo, S., Lee, K. J., Kim, B. J., Koh, W. J., . . . Park, Y. K. (2013). Molecular genetics of Mycobacterium tuberculosis resistant to aminoglycosides and cyclic peptide capreomycin antibiotics in Korea. *World J Microbiol Biotechnol, 29*(6), 975-982. doi: 10.1007/s11274-013-1256-x
5. Mathys, V., Wintjens, R., Lefevre, P., Bertout, J., Singhal, A., Kiass, M., . . . Bifani, P. (2009). Molecular genetics of para-aminosalicylic acid resistance in clinical isolates and spontaneous mutants of Mycobacterium tuberculosis. *Antimicrob Agents Chemother, 53*(5), 2100-2109. doi: 10.1128/AAC.01197-08

6. Koser, C. U., Bryant, J. M., Becq, J., Torok, M. E., Ellington, M. J., Marti-Renom, M. A., . . . Peacock, S. J. (2013). Whole-genome sequencing for rapid susceptibility testing of M. tuberculosis. *N Engl J Med, 369*(3), 290-292. doi: 10.1056/NEJMc1215305
7. Manson, A. L., Cohen, K. A., Abeel, T., Desjardins, C. A., Armstrong, D. T., Barry, C. E., 3rd, . . . Earl, A. M. (2017). Genomic analysis of globally diverse Mycobacterium tuberculosis strains provides insights into the emergence and spread of multidrug resistance. *Nat Genet, 49*(3), 395-402. doi: 10.1038/ng.3767
8. , A., Strong, M., Muthukrishnan, P., Weiner, B. K., Church, G. M., & Murray, M. B. (2009). Tuberculosis drug resistance mutation database. *PLoS Med, 6*(2), e2. doi: 10.1371/journal.pmed.1000002
9. Zheng, J., Rubin, E. J., Bifani, P., Mathys, V., Lim, V., Au, M., . . . Camacho, L. R. (2013). para-Aminosalicylic acid is a prodrug targeting dihydrofolate reductase in Mycobacterium tuberculosis. *J Biol Chem, 288*(32), 23447-23456. doi: 10.1074/jbc.M113.475798
10. Zaunbrecher, M. A., Sikes, R. D., Jr., Metchock, B., Shinnick, T. M., & Posey, J. E. (2009). Overexpression of the chromosomally encoded aminoglycoside acetyltransferase eis confers kanamycin resistance in Mycobacterium tuberculosis. *Proc Natl Acad Sci U S A, 106*(47), 20004-20009. doi: 10.1073/pnas.0907925106
11. Dheda, K., Gumbo, T., Maartens, G., Dooley, K. E., McNerney, R., Murray, M., . . . Warren, R. M. (2017). The epidemiology, pathogenesis, transmission, diagnosis, and management of multidrug-resistant, extensively drug-resistant, and incurable tuberculosis. *Lancet Respir Med*. doi: 10.1016/S2213-2600(17)30079-6
12. Bloemberg, G.V., Gagneux, S., Böttger, E.C. (2015). Acquired Resistance to Bedaquiline and Delamanid in therapy for tuberculosis. N Engl J Med (373):1986-8
13. Zhang, S., Chen, J., Cui, P., Shi, W., Zhang, W., & Zhang, Y. (2015). Identification of novel mutations associated with clofazimine resistance in Mycobacterium tuberculosis. *J Antimicrob Chemother, 70*(9), 2507-2510

Table S2: Marginal likelihood estimates from Bayesian statistical analysis to test the relaxed molecular clock under GTR model for comparison of the different demographics

Demographic model	Path sampling	Stepping stone	Bayes Factor
Constant	5905668	5905667	
Logistic	5905637	5905636	31
Exponential	5905636	5905634	32
Expansion	5905638	5905634	30
Bayesian Skyline	5905636	5905636	32

Figure S2: Phylogenetic tree of Daru isolates together with global representative *M. tuberculosis* genomes (labels-black). Tree constructed using 7012 single nucleotide polymorphisms (using RAxML v.7.4.2) and rooted on *Mycobacterium. bovis*. The 95 strains formed a monophyletic clade (collapse-green) among the modern Beijing sub-lineage while 5 strains were among the Euro-American lineage (labels-red and purple)

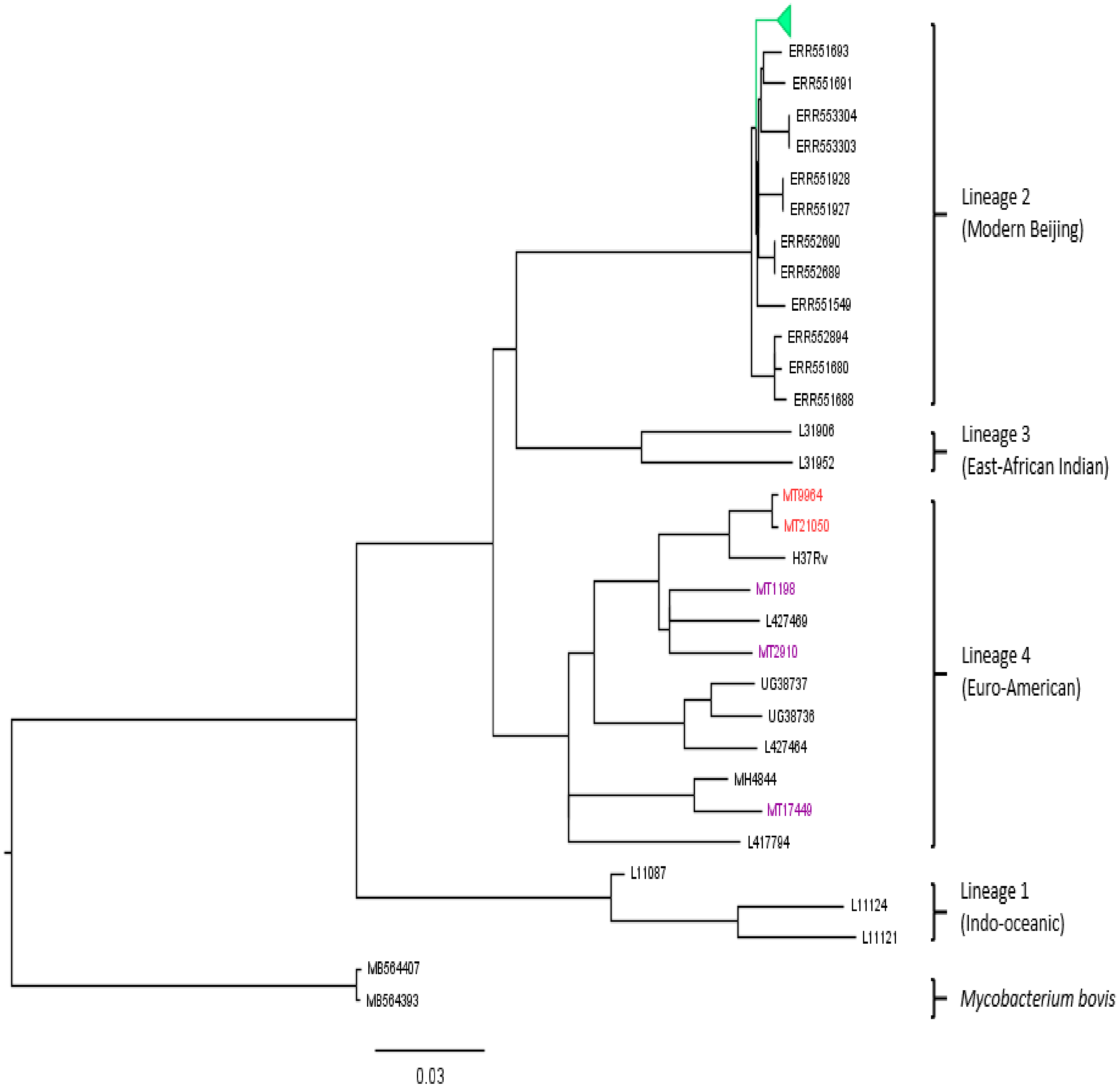

Figure S3: Pairwise SNP distance among Daru Beijing sub-lineage strains showing a unimodal distribution

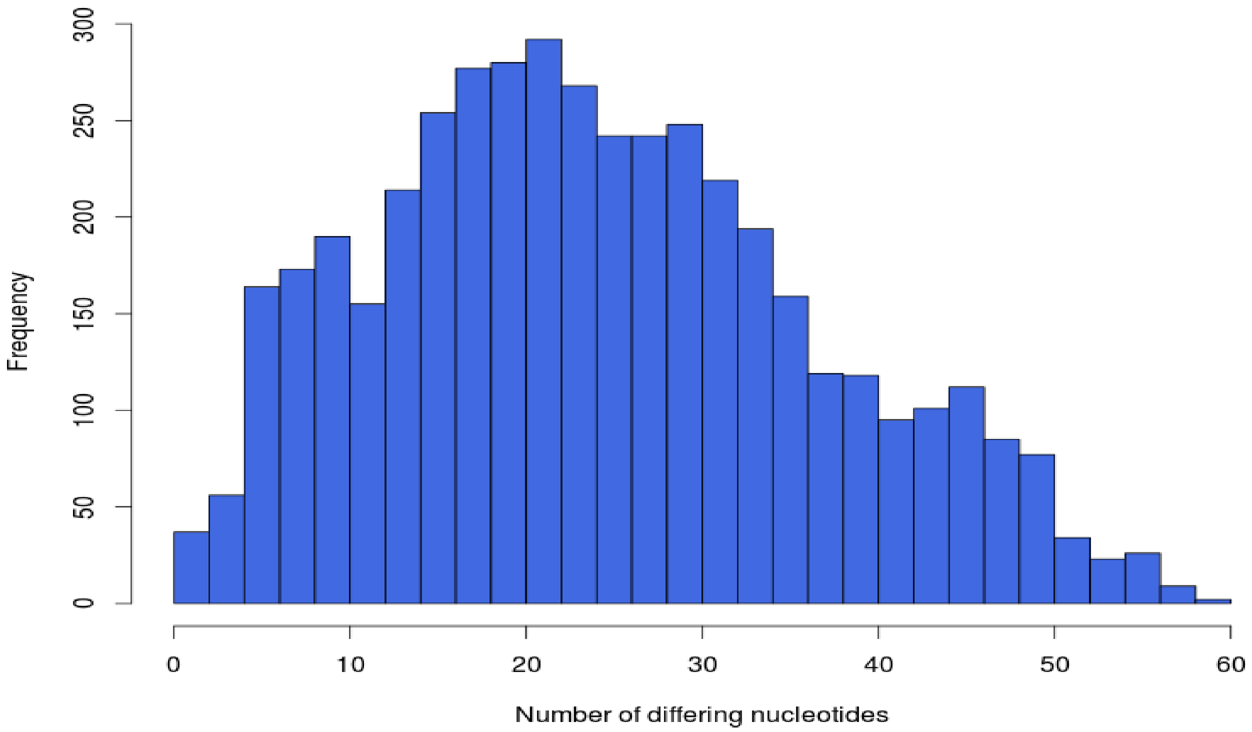

Table S3: Lineage defined SNPs according to Coll et al (Coll et al., 2014)

Beijing No	Position	Allele change	Codon change	Gene Name	Coding
Lineage 2	497491	G/A	GAC/GAT	*glnH*	Synonymous
Lineage 2.2	2505085	G/A	GCC/GCT	*cobC*	Synonymous
Lineage 2.2.1	797736	C/T	CTC/CTT	_	Synonymous
Lineage 2.2.1.1	4248115	C/T	GAC/GAT	*embB*	Synonymous

Coll, F., McNerney, R., Guerra-Assuncao, J. A., Glynn, J. R., Perdigao, J., Viveiros, M., . . . Clark, T. G. (2014). A robust SNP barcode for typing Mycobacterium tuberculosis complex strains. *Nat Commun, 5*, 4812. doi: 10.1038/ncomms5812

Table S4: Unique SNPs among Daru Beijing strains that differentiate it from the nearest neighboring Modern Beijing strain among the global representative genomes

Position	Change	Gene	Locus	Type of change	Amino Acid	Product
318517	C->T	*oplA*	Rv0266c	Non-synonymous	Ala880Thr	5-Oxoprolinase
443264	C->T		Rv0365c	Non-synonymous	Val312Met	Fructose-bisphosphate aldolase
773661	C->T	*echA4*	Rv0673	Non-synonymous	Pro180Leu	enoyl-CoA hydratase
799289	C->G		Rv0698	Non-synonymous	Arg153Gly	Predicted ideR regulon
971200	T->C	*fadE10*	Rv0873	Synonymous	phe232phe	Acyl-CoA dehydrogenase
1140664	T->G	*mfd*	Rv1020	Synonymous	Thr566Thr	Transcription-repair coupling factor
1293748	C->G	*narI*	Rv1164	Non-synonymous	Arg115Gly	Nitrate reductase
1310874	C->T		Rv1179c	Non-synonymous	Gly809Asp	
1422353	G->T		Rv1273c	Synonymous	Ala566Ala	ABC transporter ATP binding protein
1596224	C->T		Rv1421	Synonymous	Thr82Thr	Nucleotide binding protein
1820488	G->A	*cydC*	Rv1620c	Synonymous	Asp402Asp	ABC transporter ATP binding protein
2161210	G->A	*aceAa*	Rv1915	Non-synonymous	Val250Ile	Isocitrate lyase
2588998	C->G	*uspA*	Rv2316	Non-synonymous	Thr54Ser	ABC transporter permease UspA
2595702	C->G		Rv2323c	Non-synonymous	Val190Leu	
2711499	G->A		Rv2414c	Non-synonymous	Pro460Ser	
2937218	G->A		Rv2609c	Synonymous	Thr216Thr	Exppolyphosphatase
2955138	C->T		Rv2628	Synonymous	Gly27Gly	Uncharacterized protein
2974938	G->T		Rv2650	Synonymous	Arg99Arg	
3013291	G->A		Rv2696c	Synonymous	Arg106Arg	
3248028	G->A	*ppsA*	Rv2931	Non-synonymous	Val862Ile	polyketide synthase type1
3505648	C->G	*fadE24*	Rv3139	Non-synonymous	Arg96Gly	Acyl-CoA dehydrogenase
3618435	G->T	*secA1*	Rv3240c	Non-synonymous	Asp699Glu	Protein translocase subunit
3660529	C->A		Rv3277	Non-synonymous	Leu218Met	
3716646	G->A	*dacB1*	Rv3330	Synonymous	Gly290Gly	D-amyl-D-alanine carboxypeptidase
3772657	C->T		Rv3360	Stop Gained	Arg3*	Predicted-Forkhead-Associated domain
3838972	C->A	*rimI*	Rv3420c	Non-synonymous	Gly31Cys	alanine acetyltransferase
3876935	G->A	*rplQ*	Rv3456c	Synonymous	Asp166Asp	50S ribosomal protein L17
3922552	C->T	*fadE26*	Rv3504	Non-synonymous	Arg28Cys	Acyl-CoA dehydrogenase
4016415	C->T	*fad34-kstR*	Rv3573c-Rv3574	Intergenic		
4245131	G->A	*embA*	Rv3794	Synonymous	Pro633Pro	Arabinosyl transferase A
4278559	C->T		Rv3813c	Synonymous	Val219Val	
4335119	C->T	*gltB*	Rv3859c	Synonymous	Glu321Glu	Glutamate synthase

Table S5: Polymorphisms that are specific to Beijing lineage outbreak cluster Clade B, C and D

Position	Nucleotide change	Gene	Amino Acid	Type of change
635725	G->T	Rv0543c	Leu51Ile	Non-synonymous
781687	A->G	*rpsL*	Lys43Arg	Non-synonymous
844023	T->C	*fadE9*	Asp131Gly	Non-synonymous
950508	AGC->A	*pdc*	Rv0853c	frameshift
1595966	CG->C	*uvrC*	Rv1420	frameshift
1611757	C->T	Rv1433	Ser108Ser	synonymous
3015445	C->T	Rv2700	Thr81Thr	synonymous
3292835	G->C	*pks1*	Ala1173Ala	synonymous
3375035	C->T	Rv3015c	Ala210Ala	synonymous
4364046	T->C	*mycp1*	Thr238Ala	Non-synonymous

Figure S4: Sequence reads mapped of the *H37Rv* reference showing two *rpoB* mutations known to confer rifampicin resistance. The orange mutation (p.Ile480Val) is outside the 81bp rifampicin resistance determining region.

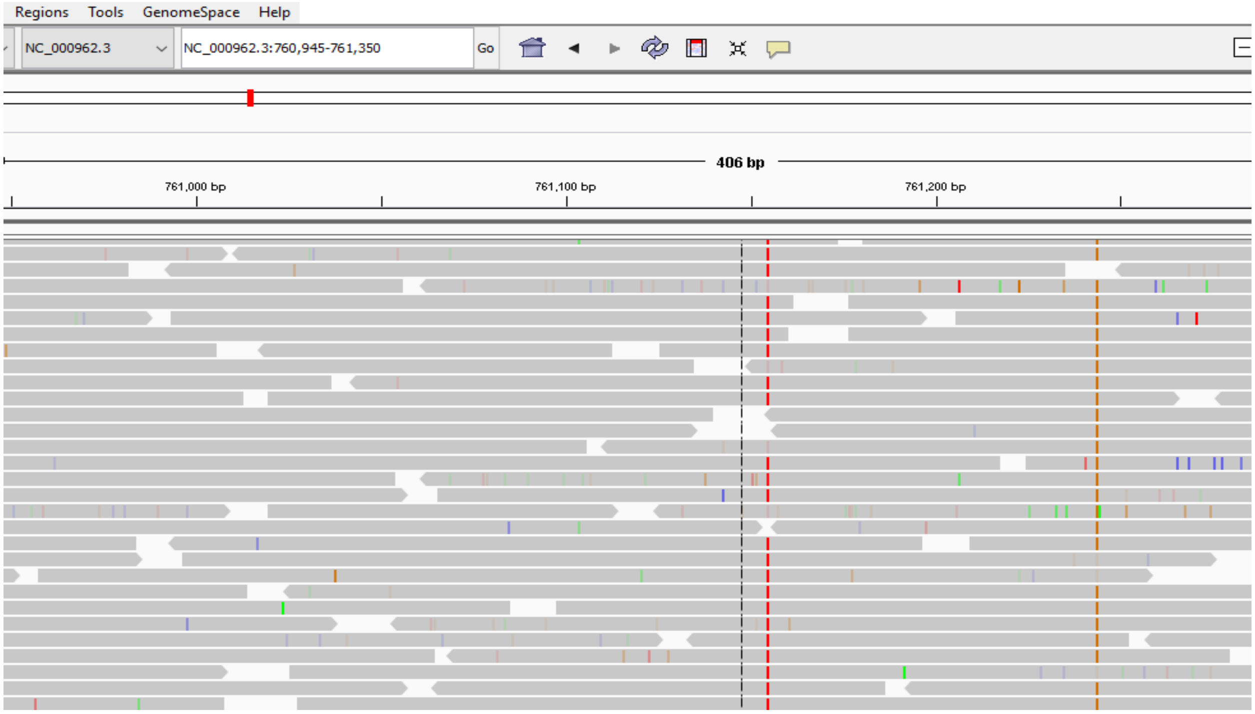

Figure S5: Sequence reads mapped on *H37Rv* reference genome spanning the *rpoB* gene (759807- 763325) but without any observable mutation at sequence depth of X75

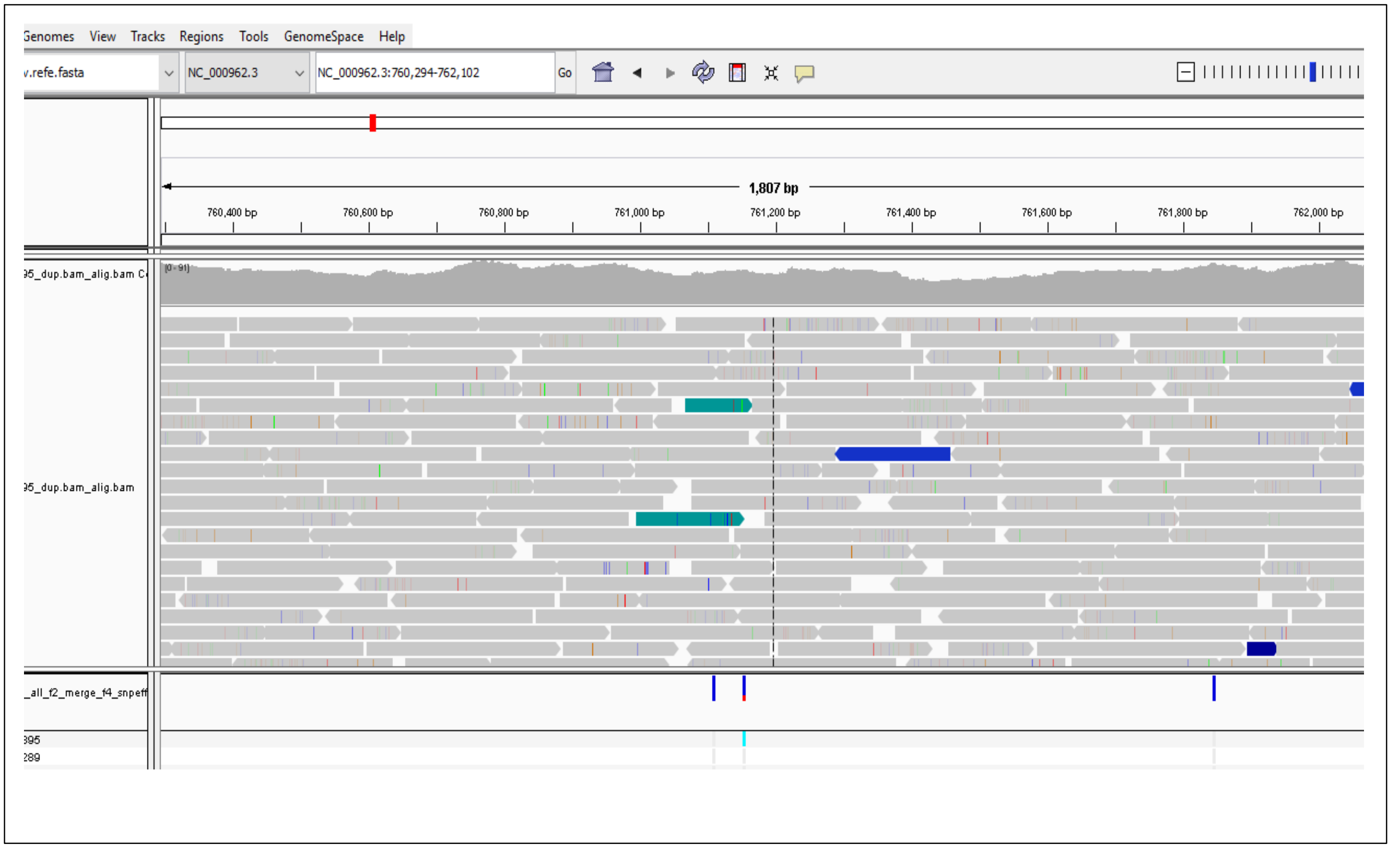

Figure S6: Sequence reads mapped onto *H37Rv* reference indicating a deletion (2287064-2291054) spanning pncA gene among two pyrazinamide resistant Beijing strains

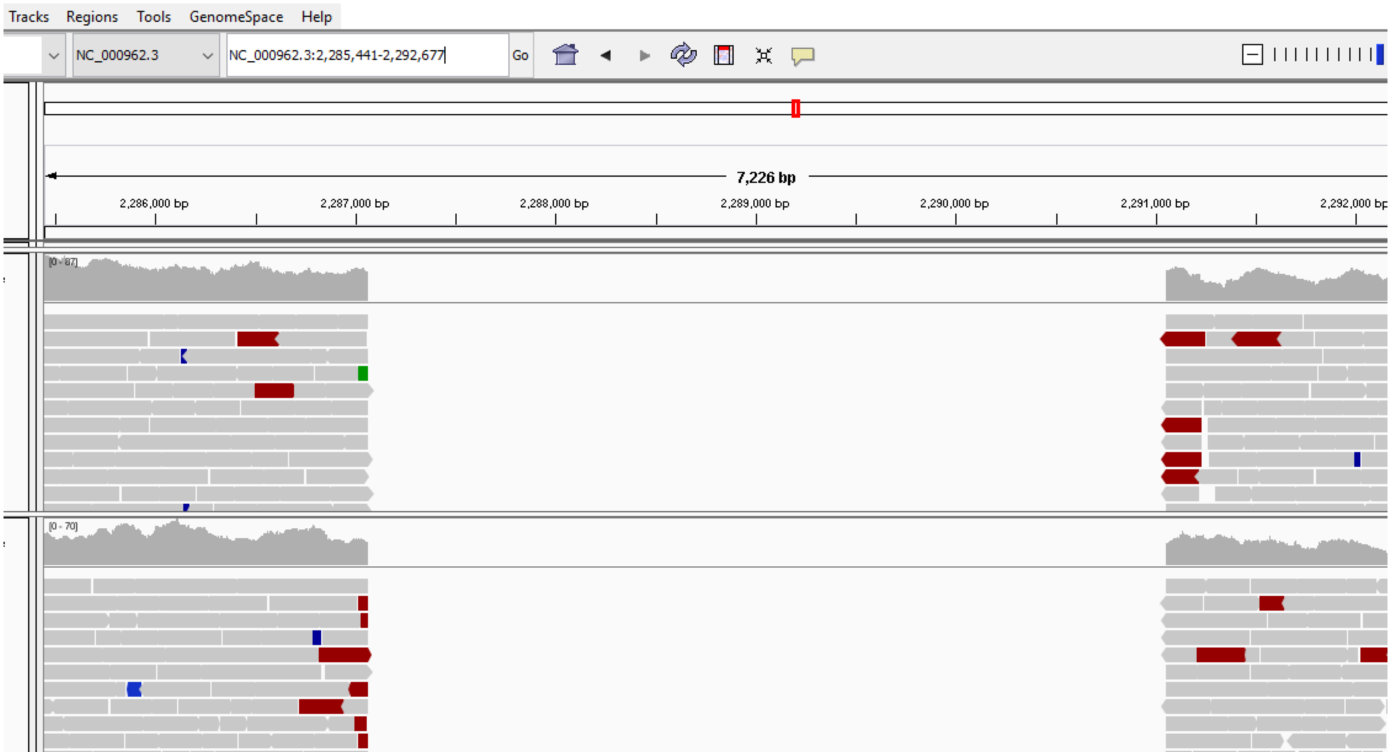

Figure S7: Interaction of genes with SNPs known to confer XDR resistance among Beijing sub-lineage strains

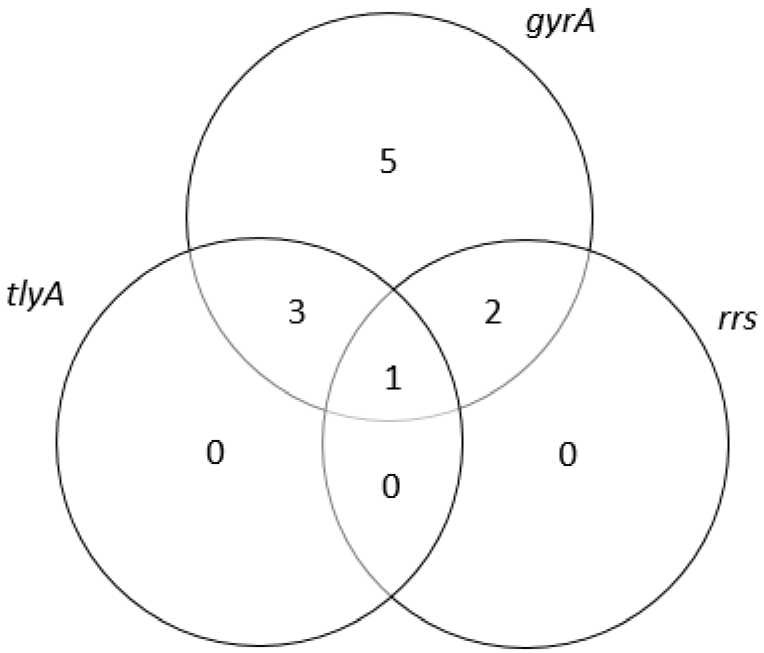

Figure S8: Transmission networks constructed using SeqTrack for clades C and D, showing the SNP distances (branch numbers). Each circle represents an isolate while larger ones represent two or three isolates with similar sequences. Green circle/blue outline-MDR, green circle/red outline-pre-XDR, red circle-XDR and blue circle-susceptible isolates. Dashed shaped lines represent identified clusters of directly linking isolates

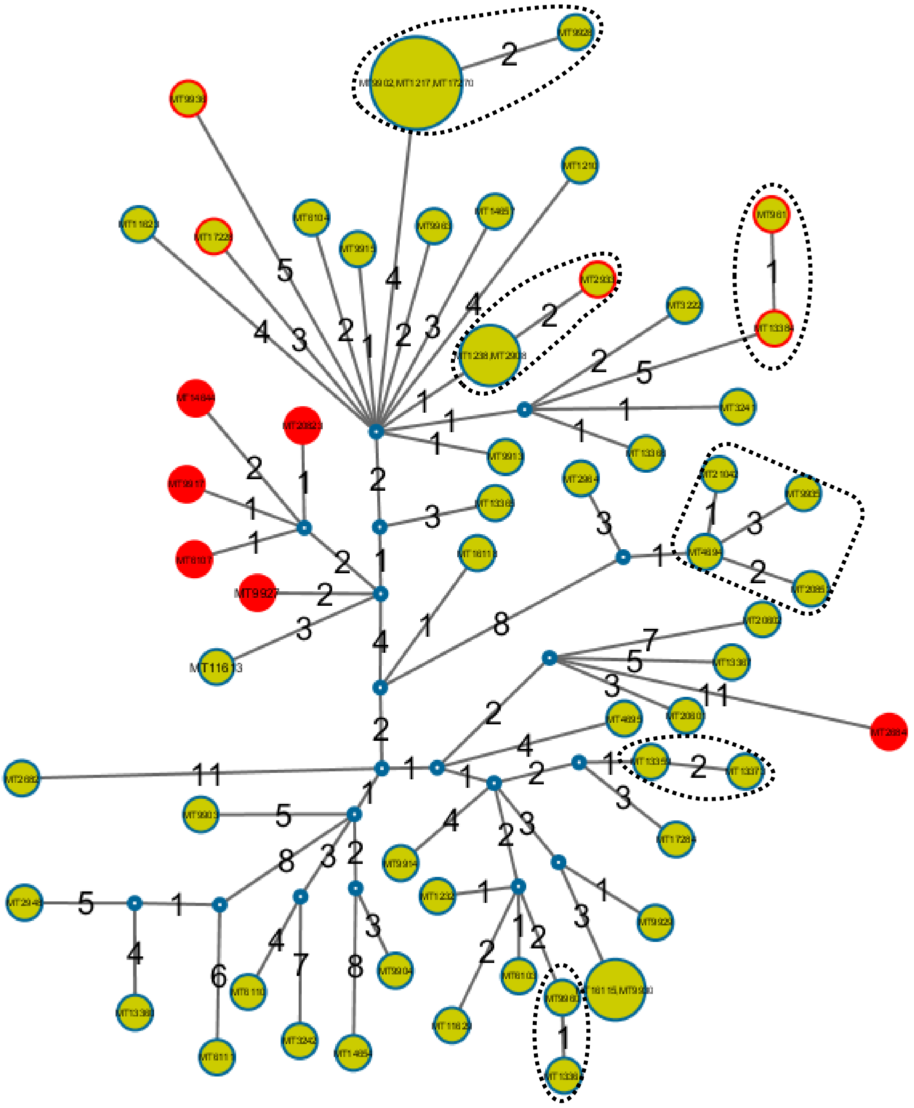

Clade C

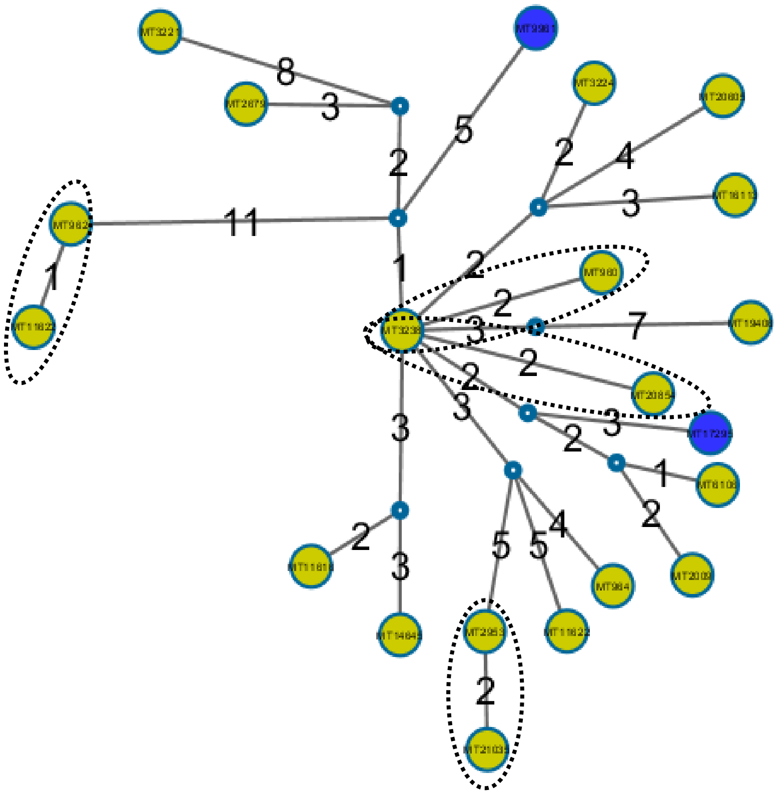
MT3221
MT2679
MT3224
MT980
MT3238
MT20854
MT17295
MT984
MT2953
MT21035

Clade D
